# Single-Cell Analysis of Aneurysmal Aortic Tissue in Patients with Marfan Syndrome Reveals Dysfunctional TGF-β Signaling

**DOI:** 10.3390/genes13010095

**Published:** 2021-12-30

**Authors:** Ashley Dawson, Yanming Li, Yang Li, Pingping Ren, Hernan G. Vasquez, Chen Zhang, Kimberly R. Rebello, Waleed Ageedi, Alon R. Azares, Aladdein Burchett Mattar, Mary Burchett Sheppard, Hong S. Lu, Joseph S. Coselli, Lisa A. Cassis, Alan Daugherty, Ying H. Shen, Scott A. LeMaire

**Affiliations:** 1 Division of Cardiothoracic Surgery, Michael E. DeBakey Department of Surgery, Baylor College of Medicine, Houston, TX 77030, USA; Ashley.E.Dawson@uth.tmc.edu (A.D.); yanming.li@bcm.edu (Y.L.); Yang.Li@bcm.edu (Y.L.); pren1228@gmail.com (P.R.); hernan.vasquez@bcm.edu (H.G.V.); chen.zhang@bcm.edu (C.Z.); Kimberly.Rebello@bcm.edu (K.R.R.); Waleed.Ageedi@bcm.edu (W.A.); jcoselli@bcm.edu (J.S.C.); hyshen@bcm.edu (Y.H.S.); 2 Department of Cardiovascular Surgery, Texas Heart Institute, Houston, TX 77030, USA; aazares@texasheart.org; 3 Division of Cardiothoracic Transplantation and Circulatory Support, Baylor College of Medicine, Houston, TX 77030, USA; aladdein.mattar@bcm.edu; 4 Saha Cardiovascular Research Center, University of Kentucky, Lexington, KY 40536, USA; mary.sheppard@uky.edu (M.B.S.); hlu4@uky.edu (H.S.L.); adaugh@uky.edu (A.D.); 5 Department of Physiology, University of Kentucky, Lexington, KY 40536, USA; 6 Department of Pharmacology and Nutritional Sciences, University of Kentucky, Lexington, KY 40536, USA; lcassis@uky.edu

**Keywords:** aneurysm, smooth muscle cell differentiation, Marfan syndrome, molecular biology

## Abstract

The molecular and cellular processes leading to aortic aneurysm development in Marfan syndrome (MFS) remain poorly understood. In this study, we examined the changes of aortic cell populations and gene expression in MFS by performing single-cell RNA sequencing (scRNA seq) on ascending aortic aneurysm tissues from patients with MFS (*n* = 3) and age-matched non-aneurysmal control tissues from cardiac donors and recipients (*n* = 4). The expression of key molecules was confirmed by immunostaining. We detected diverse populations of smooth muscle cells (SMCs), fibroblasts, and endothelial cells (ECs) in the aortic wall. Aortic tissues from MFS showed alterations of cell populations with increased de-differentiated proliferative SMCs compared to controls. Furthermore, there was a downregulation of *MYOCD* and *MYH11* in SMCs, and an upregulation of *COL1A1/2* in fibroblasts in MFS samples compared to controls. We also examined TGF-β signaling, an important pathway in aortic homeostasis. We found that *TGFB1* was significantly upregulated in two fibroblast clusters in MFS tissues. However, TGF-β receptor genes (predominantly *TGFBR2*) and *SMAD* genes were downregulated in SMCs, fibroblasts, and ECs in MFS, indicating impairment in TGF-β signaling. In conclusion, despite upregulation of *TGFB1*, the rest of the canonical TGF-β pathway and mature SMCs were consistently downregulated in MFS, indicating a potential compromise of TGF-β signaling and lack of stimulus for SMC differentiation.

## 1. Introduction

Marfan syndrome (MFS), an autosomal dominant disease, is associated with aortic aneurysms predominantly involving the aortic root [1]. MFS is caused by mutations in the gene encoding the glycoprotein fibrillin-1 (*FNB1*) [2], which are believed to disrupt cell signaling and vascular integrity [3,4]. Patients with MFS have altered smooth muscle cell (SMC) phenotypes. Studies have indicated changes in the expression of contractile and extracellular matrix (ECM) genes [5,6] and alterations in SMC appearance [7]. New sequencing technologies, such as single-cell RNA (scRNA) sequencing, have provided a more precise understanding of the range of SMC phenotypes [8].

We used scRNA sequencing to better understand the non-immune cell populations in the aortic wall in MFS. Here, we focused on non-immune cells because they are in direct contact with fibrilin-1 and are the predominant permanent cells forming the aortic wall, making them likely drivers of aortic pathology in MFS. We hypothesized that, compared with control cells, non-immune cells in aneurysmal tissue from patients with MFS would show changes in phenotype and cell-specific gene expression that are detectable by single-cell transcriptome analysis.

## 2. Materials and Methods

### 2.1. Human Tissue Samples

The protocol for collecting human tissue samples was approved by the institutional review board at Baylor College of Medicine. Written informed consent was provided by all subjects or their legal representatives before enrollment. All experiments conducted with human tissue samples were performed in accordance with the relevant guidelines and regulations and conformed to the principles outlined by the Declaration of Helsinki.

Diseased tissue samples were obtained from patients (Supplementary Table S1) with advanced aortic root and ascending aortic aneurysms (without dissection) who were diagnosed with MFS according to the revised Ghent criteria, which do not require a known mutation of *FBN1* for diagnosis. We excluded patients taking angiotensin II receptor blockers and those with other genetic conditions related to aortopathy, such as bicuspid aortic valve, and patients with ascending aortic dissection, aortitis, or active infection. For controls, non-aneurysmal ascending aortic tissue samples were obtained from age-matched heart transplant donors and recipients. Total sample number for all single-cell experiments was 4 controls and 3 patients with MFS. The median age was 38 years (range, 23–40 years) for MFS patients and 41 years (range, 23–47 years) for controls.

### 2.2. Antibodies

The TGF-β antibody (#3709), TGF-β receptor II antibody (#41896), Phopho-SMAD2 (Ser465/Ser467) antibody (#18338), SMAD2 antibody (#5339), and cyclin D1 (#2978) antibody were obtained from Cell Signaling. The α-smooth muscle Actin (ab89989) antibody was purchased from Abcam.

### 2.3. Tissue Processing

Before processing, pieces of unfixed aortic tissue were cut and embedded in optimal cutting temperature (OCT) compound for immunofluorescence staining. For single-cell analysis, each sample was placed on ice, and a 1–2 cm piece of aortic tissue was separated into adventitial, medial, and intimal layers and minced into small pieces in Hank’s balanced salt solution (HBSS) (without Ca^2+^ and Mg^2+^) with 10% FBS. The tissue was placed in an enzyme cocktail prepared with 3 mg/mL collagenase type II, 0.15 mg/mL collagenase type XI, 0.25 mg/mL soybean trypsin inhibitor, 0.1875 mg/mL elastase lyophilized, 0.24 mg/mL hyaluronidase type I, and 2.38 mg/mL HEPES in HBSS (with Ca^2+^ and Mg^2+^) and digested in a 37 °C water bath for 1–2 h depending on the tissue dissociation (confirmed microscopically). Medial tissues, because of longer digestion times, were digested separately from the adventitial and intimal layers. A 40-μm cell strainer was used to remove debris from the samples, and the flow-through from the medial and adventitial/intimal layers was recombined and centrifuged at 300× *g* for 10 min. The pellet was re-suspended in HBSS (without Ca^2+^ and Mg^2+^) with 5% FBS and incubated on ice for 30 min. Cells were stained with DAPI, and flow cytometry was used to collect live single cells.

### 2.4. Single-Cell RNA Sequencing and Raw Data Processing

Single-cell suspensions were processed in the 10× Genomics Chromium system, and 3′ Gene Expression v3 Libraries were constructed and sequenced using the Illumina NovaSeq 6000 system. Raw fastq data alignment was processed by using the Cell Ranger 3.0 with GRCh38 as the reference genome. Unique reads mapped to the reference genome were tagged with unique molecular identifiers (UMIs), and a UMI count matrix was formed. The remainder of the analysis was performed using R and associated packages [9,10,11,12,13,14,15]. The data were processed by R package Seurat [13] with the following quality control criteria: gene count per cell >200 and <4000 (or 5000 depending on the overall average level of genes per cell in each sample) and percentage of mitochondria genes <10%. Additionally, we identified a high level of *HBB* expression in our data set, which likely indicated red blood cell contamination. We filtered the cells to include only those without expression of the HBB gene. Individual sample non-immune cell data, cell counts from each sample comprising each cluster, and quality control metrics within the non-immune cells in each sample are shown in Supplementary Materials online, Figure S1A–C, respectively. Control tissue contributed more SMCs to the populations and a higher feature expression/cell than did patient tissue.

### 2.5. Cell Clustering and Identification

The data were normalized, scaled, and subjected to linear dimensional reduction, clustering, and non-linear dimensional reduction for t-distributed stochastic neighbor embedding (t-SNE) visualization as described in the Seurat vignettes. To combine multiple datasets and ensure that no non-immune cells were missed, we extracted the UMI counts for the non-immune cells from each sample and recombined them by using a resolution of 0.6. Conserved (marker) genes for each cluster were identified by the function “FindAllMarkers” in Seurat [13]. The general cell type of each non-immune cell cluster was further identified using known markers. Feature expression per cluster and overall expression in MFS compared to control tissues are shown in Supplementary Figure S2.

### 2.6. Differential Gene Analysis and Function Annotation

Differential gene expression within each cluster between MFS and control samples was determined using WaVE-EdgeR [16,17,18]. Differential gene expression between groups of cells (e.g., SMCs) and overall differential gene expression between MFS and control samples were identified using the “Find Markers” function in Seurat [19].

### 2.7. Module (Composite) Score

The module score for a particular list of genes expressed in each cluster was calculated by using the function “AddModuleScore” in Seurat. To avoid bias by selecting only certain genes, we compiled set lists (e.g., all collagen or all integrin genes) for use in the cluster analyses. All genes in each list were used in the module score unless they were not expressed in any clusters.

### 2.8. Correlation Analysis

To identify genes correlating with a particular gene, we performed a correlation analysis for the gene of interest within each cluster of interest using Spearman’s correlation in R.

### 2.9. Immunofluorescence

OCT-embedded aortic sections were sliced into 5-μm sections onto slides. Tissues were fixed with BD Cytofix/Cytoperm (BD Biosciences, San Jose, CA, USA) and permeabilized with 1× BD Perm/Wash (BD Biosciences). Nonspecific staining was reduced by blocking with 10% donkey serum for 1 h. The sections or cells were then incubated with primary antibody at 4 °C overnight, washed with 1× BD Perm/Wash, and incubated with secondary antibody. Antibodies used for immunostaining are listed in Supplementary Table S2. Nuclei were counterstained with 4’,6-diamidino-2-phenylindole (DAPI). The slides were mounted with ProLongTM Diamond Antifade Mountant with DAPI (Life Technologies, Corporation, Eugene, OR, USA). Sections incubated with secondary antibody alone served as negative controls. Fluorescence microscopy at 10× and 20× was performed (Leica microscope, Leica Microsystems Inc., Buffalo Grove, IL, USA) using the same exposure times for each wavelength across all samples. Fiji/ImageJ [20,21] imaging was used to quantify three different areas for each staining of each sample (three controls, three patients with MFS). For dual antibody staining, co-localization was identified as falling within a set yellow threshold (red/green overlap), the bounds of which were kept constant throughout the sample analyses. Proliferative SMCs (identified by co-localization of CCND1 and SM22α) were quantified by multiplying the total area of colocalization by the mean brightness value. Representative images were taken from the samples that provided the median values on immunofluorescence quantification. Confocal microscopy was performed using the Leica SP5 confocal microscope.

## 3. Results

### 3.1. Overall Cell Populations and Non-Immune Cells in the Aortic Wall

**Figure 1:**
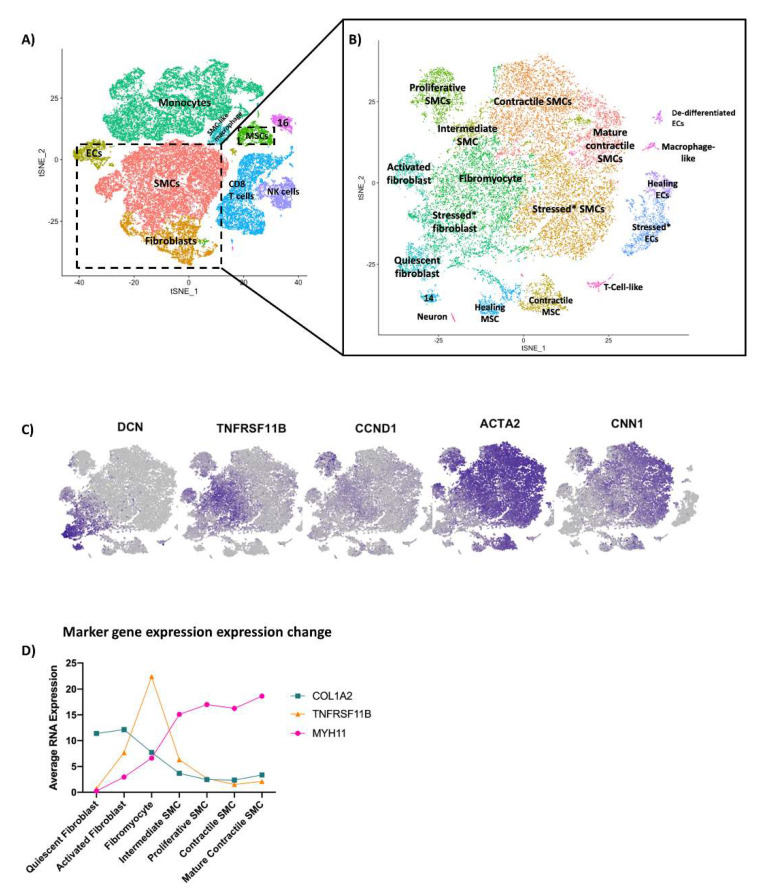
Heterogeneity of non-immune cells in the aortic wall in combined samples of 4 controls and 3 patients with MFS. (**A**) General immune and non-immune cell types in the aortic wall. Cell cluster proportion represents the number of cells after digestion and processing and likely does not represent the true cell proportion in the aortic wall. (**B**) Non-immune cells were extracted from the data and recombined. (**C**) Visualization of the change in expression of common marker genes across the phenotypic continuum of SMCs and fibroblasts. (**D**) Change in expression of *MYH11* (contractile marker), *COL1A2* (ECM marker), and *TNFSRF11B* (fibromyocyte marker) throughout the phenotypic continuum in SMCs and fibroblasts.

To create a comprehensive catalog of cell clusters, we first combined the data from MFS (*n* = 3) and control (*n* = 4) samples (Figure 1A). We identified major cell groups in the aortic wall by using known markers (Supplementary Material online, Figure S3) for SMCs (*SMTN*, *MYH11*), fibroblasts (collagen genes, *LUM*, *DCN*), endothelial cells (ECs) (*PECAM11*, *POSTN*, *VWF*), monocytes and macrophages (*CD14*, *CD68*), CD8 T cells (*CD8A*, *CD8B*), natural killer cells (*KLRC1*), and mast cells (*CPA3* and *TPSB2*). Cluster 16 was consistent for both CD8 T cells and natural killer cells and was left unnamed in this analysis.

Samples were evaluated independently using the Seurat package in R, and non-immune cells in each sample were extracted and re-combined (Figure 1B), showing 18 clusters. Supplementary Figure S3B shows the expression of well-known markers within each cluster. Six clusters highly expressing contractile genes, namely *ACTA2*, *TAGLN*, *CALD1*, *TPM2*, *MYH11*, *SMTN*, *CNN1*, and *MYOCD*, were classified as SMC-like. Three clusters highly expressing ECM component genes, including *LTBP2*, *TNFRSF11B*, *CYTL1*, *COL6A1*, *COL1A1*, *COL1A2*, *COL5A1*, *LTBP1*, *BGN*, and *FN1*, were classified as fibroblasts. We classified three clusters highly expressing *PECAM11*, *POSTN* and *VWF* as ECs. Two clusters highly expressing *THY1*, *PDGFRB*, and *RERGL* and genes involved in angiogenesis and remodeling, such as *VEGFA*, *NOTCH3*, *ADAMTS1*, and *FABP4*, were classified as mesenchymal stem cells (MSCs), alternatively named pericytes in other studies [22].

Additionally, we identified two small clusters highly expressing genes associated with immune cells (MHC-II genes, complement-factor genes, *CD68*, *CD69*, and *CD96*). Gene expression patterns within these clusters showed macrophage-like and T-cell-like clusters.

### 3.2. Heterogeneous SMC Population in the Aortic Wall

Further analysis was conducted within SMC clusters of the combined MFS and control samples to determine SMC phenotype (Supplementary Figure S4).

#### 3.2.1. Mature Contractile and Contractile SMCs

Within these clusters, we first identified SMCs with the most traditional contractile phenotype using known markers of differentiated contractile SMCs [23]. We calculated a composite score of average RNA expression of *SMTN*, *MYH11*, and *CNN1* in each of the SMC and fibroblast clusters (Supplementary Figure S4A). The cluster with the highest expression of these markers was identified as the mature contractile SMC cluster. The phenotypes of other SMC clusters were identified by calculating the differential gene expression between each SMC-like cluster and the mature contractile SMC cluster (Supplementary Figure S4B). One cluster was similar to the mature contractile SMC cluster with few comparatively upregulated or downregulated genes. This cluster expressed high levels of contractile genes but had a slightly decreased expression of maturity markers, including *SMTN* and *CNN1*, and was named contractile SMCs.

#### 3.2.2. Stressed SMCs

One cluster had increased expression of genes involved in stress responses. Many of these genes, including *JUN*, *JUNB*, *FOS*, *FOSB*, *EGR1*, *ATF3*, *SOCS3*, *NFKBIA*, *HSPA1A*, and *HSPA1B*, have been identified as early response genes stimulated by the tissue digestion process [24]. This cluster, named stressed SMCs, may represent contractile SMCs that have shifted to a stressed state due to tissue processing procedures rather than a true separate phenotype.

#### 3.2.3. Fibromyocytes and Intermediate SMCs

One cluster expressed both contractile and ECM genes (Supplementary Material online, Figure S4C) but was more closely related to the other SMCs on correlation analysis (Supplementary Figure S4D). This cluster highly expressed *TNFSRF11B* (Supplementary Figure S4E), which encodes osteoprotegerin, a tumor necrosis factor receptor involved in vascular calcification [25]. This increased expression correlates with a similar cluster of modulated SMCs described by Wirka and colleagues [22], termed fibromyocytes to emphasize differentiation from a SMC rather than fibroblast lineage. Another cluster also highly expressed *TNSFRF11B* and genes involved in ECM structures (*ELN*, *FN1*, *VCAN*, *KRT1*) and angiogenesis (*ENG*). This cluster, classified as intermediate SMCs, expressed higher levels of contractile genes than fibromyocytes.

#### 3.2.4. De-Differentiated, Proliferative SMCs

Finally, one cluster had moderate levels of contractile genes (*MYH11*) and fibroblast genes (*CYTL1*) and increased expression of cyclin genes (predominately cyclin D) (Supplementary Figure S4F). Despite a relatively low overall gene expression compared to other clusters (Supplementary Figure S2A), the presence of this cluster in each sample (Supplementary Figure S1A) after applying quality control metrics and excluding cells expressing the contaminant *HBB* supports a true SMC cluster, rather than a cluster due to technical processing. Compared with other SMCs, this cluster had relatively increased expression of *THY1*, a stem cell marker, and increased inflammatory and endothelial markers (Supplementary Figure S4G,H). This supported a de-differentiated, proliferative SMC phenotype.

### 3.3. Fibroblast Phenotypes in the Aortic Wall

Next, we identified differentially expressed genes (DEGs) between each fibroblast cluster and the other two fibroblast clusters (Supplementary Figure S5A).

#### 3.3.1. Stressed Fibroblasts

Similar to our findings with SMCs, one cluster of fibroblasts had increased expression of genes involved in the stress response (*CXCL2*, *CXCL3*, and *IER3*) and a higher stress response module score of *ATF3*, *JUN*, *JUNB*, *FOS*, and *FOSB* (Supplementary Figure S5B). This cluster, called stressed fibroblasts, likely reflects the technical artifacts of tissue processing.

#### 3.3.2. Quiescent Fibroblasts

One fibroblast cluster had increased expression of genes associated with ECM maintenance and genes involved in fibroblast quiescence [26,27], including complement factors *C1R* and *C1S* and ECM genes *LUM* and *DCN*. These cells also expressed increased levels of *SERPINF1*, an angiogenesis inhibitor [28]. This was consistent with quiescent fibroblasts, which synthesize and maintain the ECM but can be stimulated to re-enter the cell cycle to proliferate and promote healing. The quiescent fibroblasts had increased composite scores for genes involved in ECM remodeling including matrix metalloproteinases and A Disintegrin and Metalloprotease with Thrombospondin motifs (*ADAMTS* genes) (Supplementary Figure S5C). Several enzymes in these groups have been linked to aortic aneurysm development. Previously, we showed that ADAMTS-4 contributes to sporadic ascending aortic aneurysm development in mice [29].

#### 3.3.3. Activated Fibroblasts

The final fibroblast cluster had increased expression of genes involved in SMC contraction (*MYL9*, *TAGLN*, and *ACTA2*) and blood vessel development (*NOTCH3*, *THBS2*, *COL18A1*, *IGFB5*, *IGFBP5*, *PDGFRB*, and *TIMP1*). Activated fibroblasts differed from fibromyocytes. Activated fibroblasts expressed fewer contractile markers than fibromyocytes (Supplementary Figure S4A) and were closely related to other fibroblasts on correlation analysis (Supplementary Figure S4D). This cluster was consistent with activated fibroblasts, which represent proliferative fibroblasts that secrete collagen and pro-angiogenesis molecules in response to vascular injury [28,30,31,32]. Consistent with this proliferative and healing phenotype, activated fibroblasts had the highest composite scores of cyclin D genes, collagen genes, contractile genes, chemokine genes, and integrin genes when compared to other fibroblasts (Supplementary Figure S5D). The expression of *NOTCH3*, a receptor gene involved in SMC differentiation and blood vessel development [19,33], was highest in activated fibroblasts when all fibroblasts and SMCs were compared (Supplementary Figure S5E).

### 3.4. Endothelial Cells and MSCs in the Aortic Wall

We identified three clusters of ECs and calculated DEGs between them (Supplementary Figure S6A). The largest EC cluster expressed increased *POSTN* (periostin) and quiescence markers, including *IL33* and *CDKN1A*, and was highest in expressing genes involved in innate immune responses. Together, this indicates a baseline EC population. However, these cells showed the same upregulation of stress response genes, such as *ATF3*, *JUN*, *JUNB*, *FOS*, and *FOSB*, seen in SMCs and fibroblasts as indicated in the composite score (Supplementary Figure S6B). This increased expression of stress genes is likely due to tissue processing. Overall, this cluster likely represents an artificial stress response in a normal EC population.

The second largest EC cluster showed increased expression of genes involved in angiogenesis (*FLT1*, *DLL4*, *NOTCH4*, *CAV1*, and *EGFL7*) and increased composite scores of type IV collagen genes (the predominant collagen in basement membrane), integrin genes, and remodeling genes (Supplementary Figure S6C). This cluster also had the highest expression of genes involved in tight and adherens junctions (Supplementary Figure S5D), suggesting involvement in maintaining the endothelial barrier. Together, these expression patterns suggest a more activated EC type, which we named healing ECs to encompass the relatively increased expression of genes involved in angiogenesis, collagen deposition, and remodeling.

The smallest cluster had increased expression of coagulation pathway genes and an increased composite score of gap junction genes (Supplementary Figure S6E). The expression of genes encoding type I collagen, contractile proteins, and cyclin D1 was higher in this cluster (Supplementary Figure S6F), which was named de-differentiated ECs.

The two clusters of MSCs had increased expression of genes involved in angiogenesis and remodeling compared with other non-immune cells (Supplementary Figure S6G,H). One cluster had a higher expression of *THY1* and collagen genes (Supplementary Figure S6I,J) and was termed healing MSCs. The other exhibited higher expression of the contractile gene *MYH11* (Supplementary Figure S6K) and was named contractile MSCs. These two clusters may represent mesenchymal cells moving towards either a fibroblast-like, ECM-producing phenotype, or a contractile, SMC-like phenotype.

### 3.5. Immune-like Non-Immune Cells in the Aortic Wall

Despite removing immune cells from the individual samples, when non-immune samples were recombined, we identified two small clusters predominantly expressing genes associated with immune cells (Supplementary Figure S7A). One was macrophage-like with high expression of MHC-II genes and *CD14* and *CD68*. The other was T-cell-like with high expression of *CD8A* and *CD69*. These cells were not initially “captured” as immune cells in the overall data, likely due to the higher expression of non-immune cell genes than their true immune cell counterparts, and may represent cells of an immune-cell lineage that move toward a non-immune cell phenotype. Similar to SMCs, both immune-like clusters had moderate collagen I and III expression (Supplementary Figure S7B).

### 3.6. Phenotypic Continuum of SMCs and Fibroblasts

Changes in gene expression between fibroblasts and SMCs indicate a spectrum of phenotypes rather than distinct subsets. The feature plots in Figure 1C highlight the graduated transition between cell types with markers of SMC maturity (*CNN1*) and fibroblast maturity (*DCN*) expressed on either end of the continuum and markers of proliferation (*CCND1*), intermediate SMCs/fibrocytes (*TNFRSF11B*), and non-specific SMCs (*ACTA2*) expressed in the middle. The inverse relationship of the collagen I and contractile genes is represented by the expression levels of *COL1A2* and *MYH11* across the fibroblast and SMCs clusters in Figure 1D with the roughly equivalent expression of both genes corresponding to peak expression of *TNFRSF11B* in the fibromyocytes.

### 3.7. Decreased SMC Differentiation in MFS

**Figure 2:**
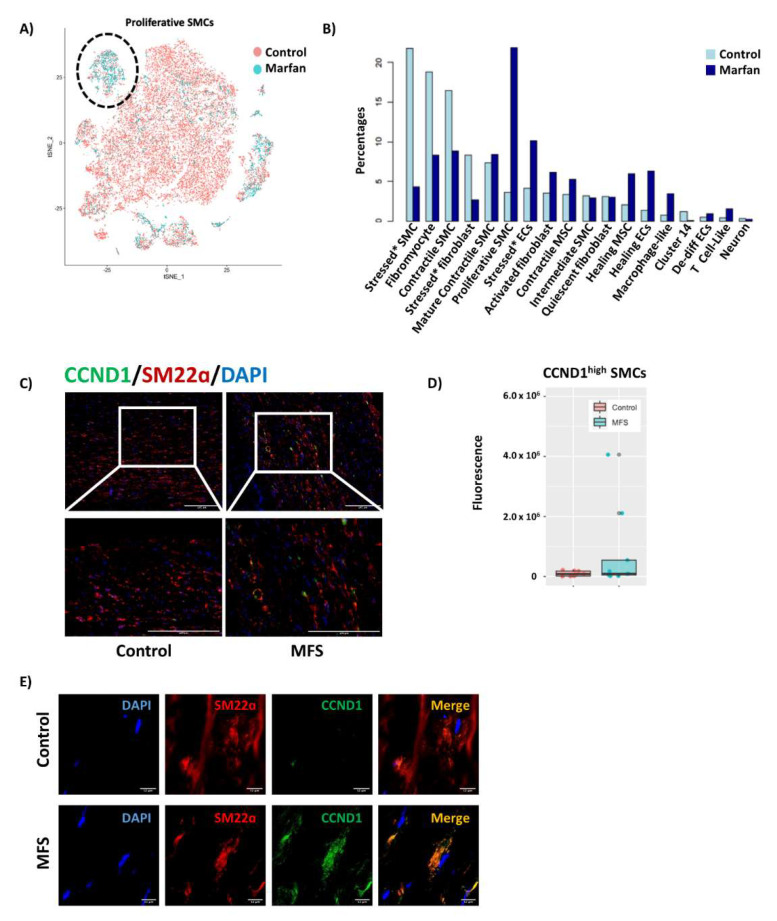
Increased proportion of de-differentiated, proliferative SMCs in MFS compared to control tissues. (**A**) Cell distribution in non-immune cells in MFS and control tissue. (**B**) Proportion of cells in each cluster in MFS and control tissues. MFS tissues had a higher proportion of de-differentiated proliferative SMCs than did control tissue. Stressed* SMCs (indicated by an asterisk) are thought to represent cells with the highest response to tissue processing rather than a true separate phenotype. (**C**) Increased CCND1^high^ de-differentiated, proliferative SMCs in MFS tissues seen on immunofluorescence, supporting our single-cell data. (**D**) Immunofluorescence quantification shows increased CCND1^high^ SMCs in MFS, consistent with increased proportion of de-differentiated, proliferative SMCs in MFS in our single-cell data as compared to control. Quantification was performed using unaltered images of 3 separate sections of slides from 3 controls and 3 patients with MFS. (**E**) Co-localization of CCND1 and SM22α on confocal microscopy.

Cell distribution in the non-immune cell population was evaluated within MFS and control tissue (Figure 2A). In MFS tissue, the largest proportion of cells was found in the de-differentiated, proliferative SMC cluster (Figure 2B), whereas this cluster was much smaller in control tissue. Consistent with the single-cell analysis, immunofluorescence studies showed increased numbers of SMCs expressing higher levels of *CCND1* in MFS tissues (Figure 2C,D).

**Figure 3:**
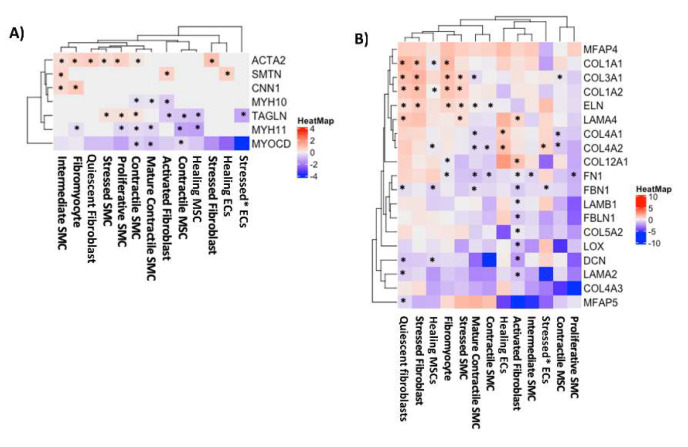
Decreased SMC differentiation in MFS compared to control. (**A**) Differential expression of contractile genes in non-immune cells in MFS compared to control tissues. (**B**) Differential expression of extracellular matrix structural genes in MFS compared to control tissues. Differential expression is visualized as log2FC with MFS compared to control. * Denotes clusters with significant differential expression (adjusted *p* < 0.05).

The differential expression of genes involved in SMC contraction is shown in Figure 3A. *ACTA2* was upregulated in SMCs in patients with MFS compared to control samples in the intermediate SMC, fibromyocyte, stressed SMC, de-differentiated proliferative SMC, and contractile SMC clusters. *SMTN* was upregulated in intermediate SMCs, and *CNN1* was upregulated in MFS in both the intermediate SMC and fibromyocyte clusters. *MYH11* was downregulated in SMCs in MFS compared to control samples in the fibromyocyte, de-differentiated proliferative SMC, contractile SMC, and mature contractile SMC clusters. *MYOCD*, a gene encoding a transcription factor that regulates multiple genes in the mature contractile SMC phenotype, was also downregulated in SMCs in MFS samples in the contractile SMC and mature contractile SMC clusters.

In the differential expression of genes involved in the ECM, *FBN1*, mutations in which cause MFS, was significantly downregulated in MFS compared to control samples in the quiescent fibroblast, healing MSC, mature contractile SMC, and activated fibroblast clusters (Figure 3B). Other structural genes, including *DCN*, *FN1*, and *FBLN1*, were also downregulated. Downregulation of many genes involved in ECM structure was consistently seen in activated fibroblasts of MFS samples. In contrast, genes encoding collagen I, collagen III, and elastin were upregulated in MFS versus control tissues, predominantly in fibromyocytes and quiescent fibroblasts.

### 3.8. Cell-Specific Expression of Canonical TGF-β Pathways among Non-Immune Cell Populations

**Figure 4:**
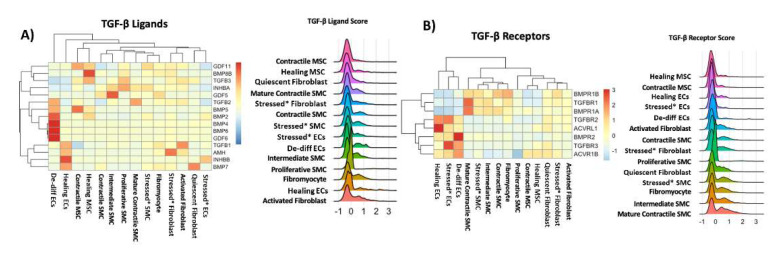
Cell-specific expression of genes in the TGF-β pathway. Expression patterns and composite score of (**A**) TGF-β ligands and (**B**) TGF-β receptors across non-immune cell clusters.

We studied the TGF-β pathway in depth because it drives SMC differentiation and is of particular interest in MFS [34]. Normally, fibrillin-1 binds and sequesters the latent TGF-β complex in the ECM. Mutations in FBN1 have been hypothesized to prevent this sequestration and increase the bioavailability of TGF-β, affecting cell proliferation and matrix deposition [35]. A list of genes involved in the TGF-β pathway was compiled using previously studied genes [36] and the most specific genes in GO enrichment pathways [37,38]. Expression patterns and composite scores of TGF-β ligand genes (Figure 4A) and receptor genes (Figure 4B) were evaluated.

Within the TGF-β ligands, TGFB1 was the most highly expressed in our dataset and was predominantly expressed in activated fibroblasts, healing ECs, and fibromyocytes. We saw consistently higher expression of bone morphogenetic protein (BMP) genes in EC clusters. When TGF-β receptor gene profiles were evaluated across all non-immune clusters, *TGFBR2* was the most highly expressed receptor and was predominantly expressed in ECs, but was present in nearly every non-immune cell cluster. SMCs additionally expressed higher levels of *TGFBR1*, *BMPR1A*, and *BMPR1B*, whereas fibroblasts expressed most receptor types.

Cells expressing higher composite TGF-β ligand scores included activated fibroblasts, healing ECs, and fibromyocytes. Cells that expressed higher TGF-β receptor scores included mature contractile SMCs, intermediate SMCs, and fibromyocytes. On junctional analysis of ligand-receptor pairs for *TGFB1*, *TGFB2*, and *TGFB3*, the highest junctional scores were between the activated fibroblasts and all EC types (Supplementary Material online, Figure S8A). Junctional analysis of BMP ligands and receptors revealed communication predominantly from the de-differentiated ECs to all other non-immune cells (Supplementary Figure S8B).

### 3.9. Compromised Canonical TGF-β Pathway in Non-Immune Cells in MFS

**Figure 5:**
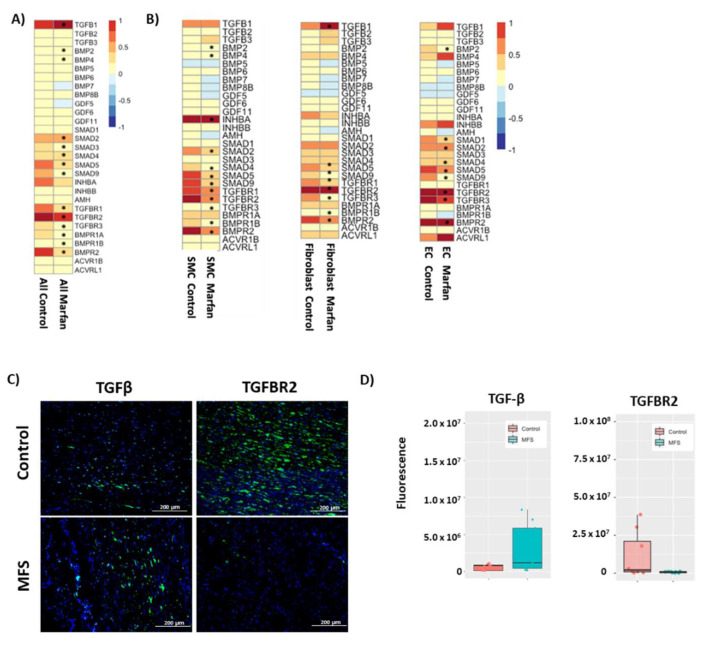
Global analysis of genes involved in the TGF-β pathway. (**A**) Differential gene expression in all combined immune and non-immune cells in MFS and control tissues. (**B**) Differential expression of genes encoding TGF-β ligands and receptors in overall cell populations in MFS and control samples in smooth muscle cells (SMCs), fibroblasts, and endothelial cells (ECs). Data are presented as RNA average. (**C**) Immunofluorescence of TGF-β1 and TGFBR2 expression throughout tissue samples, to mimic combined analysis in 4A. (**D**) Quantification of immunofluorescence results reveal increased TGFβ-1 but decreased levels of TGF-β receptor II in MFS compared to control tissues. Quantification was performed using unaltered images of 3 separate sections of slides from 3 controls and 3 patients with MFS.

We initially combined all immune and non-immune cells in MFS and control tissue to approximate the differential gene expression levels in an overall tissue analysis. When all immune and non-immune cells were combined (Figure 5A), TGFB1 was significantly upregulated in MFS compared to control tissue, whereas the TGF-β receptors and SMAD genes were downregulated in MFS samples. We then separated the data into general clusters of SMCs, fibroblasts, and ECs (Figure 5B). Overall, the gene encoding the TGF-β ligand TGFB1 was significantly upregulated in fibroblasts, and the TGF-β receptor genes and SMAD genes were downregulated in SMCs, fibroblasts, and ECs in MFS tissues. Further immunofluorescence staining showed that the TGFβ-1 was higher but TGF-β receptor II was lower in aneurysmal tissues in MFS compared to control samples (Figure 5C,D), findings consistent with our single-cell data.

**Figure 6:**
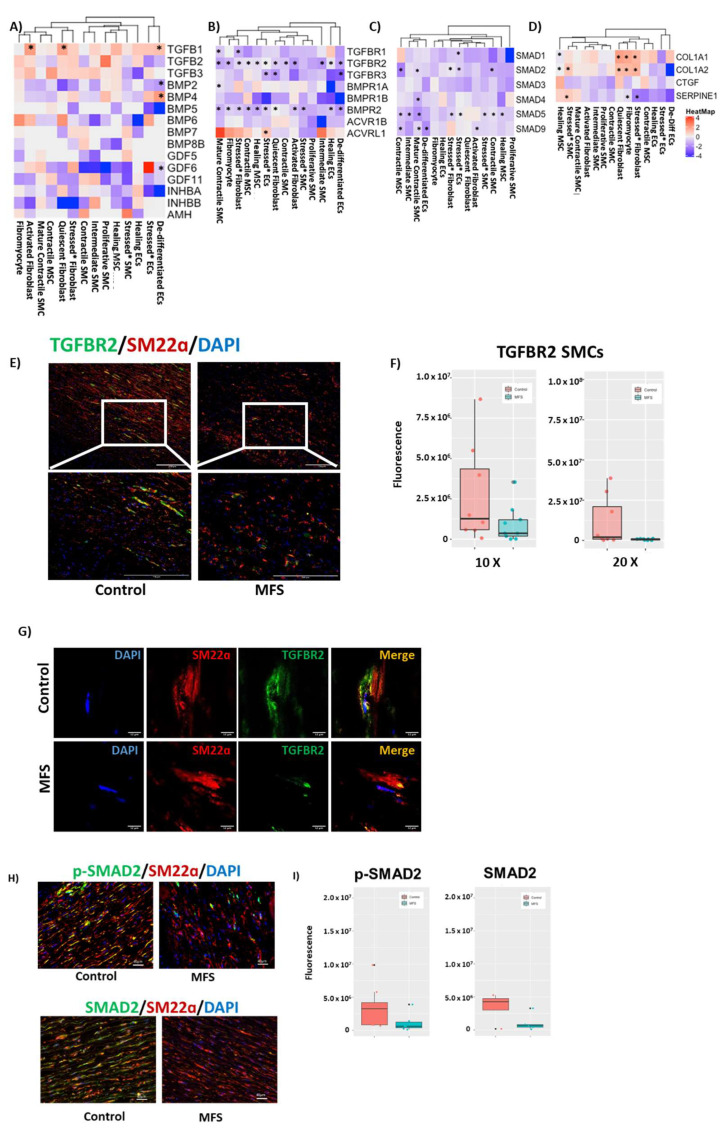
Cell-specific changes in the TGF-β pathway in MFS compared to control. (**A**–**D**) Cell-specific TGF-β pathway expression including (**A**) TGF-β ligand genes, (**B**) TGF-β receptor genes, (**C**) SMAD genes, and (**D**) target genes. * Clusters with significant differential expression (adjusted *p* < 0.05). Data are presented as log2FC in MFS compared to control. (**E**) Immunofluorescence shows decreased TGF-β receptor II levels in SMCs in MFS, supporting our single-cell data. (**F**) Immunofluorescence quantification of co-localized TGF-β receptor II and SM22α shows decreased TGF-β receptor II in SMCs in MFS, consistent with our single-cell data. Quantification was performed using unaltered images of 3 separate sections of slides from 3 controls and 3 patients with MFS. (**G**) Co-localization of TGFBR2 and SM22α on confocal microscopy. (**H**) Representative immunofluorescence images show decreased phosphorylated SMAD2 (Ser465/Ser467, p-SMAD2) and total SMAD2 (SMAD2) in SMCs in MFS. (**I**), Quantification of co-localized p-SMAD with SM22α and total SMAD2 with SM22α. Quantification was performed using unaltered images of 3 separate sections of slides from 3 controls and 3 patients with MFS. EC, endothelial cells; MSC, mesenchymal stem cells; SMC, smooth muscle cells.

Cell-specific differential expression of TGF-β ligand genes within non-immune cells was examined in MFS and control samples (Figure 6A). *TGFB1* was upregulated in MFS compared to control tissue, most predominantly in activated fibroblasts and quiescent fibroblasts. Genes that correlated with *TGFB1* in these clusters were then determined (Supplementary Tables S3 and S4). In activated fibroblasts (Supplementary Table S3), genes that correlated highly with *TGFB1* included those for proteins involved in remodeling (*MMP11*), proliferation (*PTP4A3*, *TGFBI*), angiogenesis (*PLXDC1*, *EGFL7*), ECM maintenance (*SPARC*), and degradation of misfolded glycoproteins (*DERL3*). Highly correlated genes in quiescent fibroblasts (Supplementary Table S4) included those involved in pyroptosis (*GSDMD*), ERK signaling (*WDR54*), and transcription/translation (*ZCRB1*, *EIF3L*, *HEY1*, *RPL13A*) in addition to *TGFBI*.

The non-immune cell-specific expression of genes for TGF-β receptors in MFS compared to control tissue is shown in Figure 6B. Gene expression for receptors was predominantly downregulated in MFS compared to control tissue across the different clusters. The receptor genes most consistently downregulated included *TGFBR2* and *BMPR2*. Each of these genes was downregulated in almost every cell cluster. On immunofluorescence, TGF-β receptor II levels in SMCs were also decreased (Figure 6E–G), consistent with our single-cell data.

The *SMAD* genes were downregulated in MFS compared to control across multiple cell clusters (Figure 6C). *SMAD5* was the most consistently downregulated gene including in both MSC clusters and in intermediate SMCs, mature contractile SMCs, and contractile SMCs. In the mature contractile SMC cluster, *SMAD2*, *3*, *5*, and *9* were also downregulated. Consistently, immunofluorescence staining showed that phosphorylated SMAD2 and total SMAD in SMCs of MFS patients were decreased in SMCs of MFS tissues (Figure 6H,I), indicating downregulation of TGF-β signaling.

Cell-specific expression of genes involved in the control of TGF-β signaling was also evaluated (Supplementary Figure S9). *LTBP1*, one of the components of the latent TGF-β complex, was downregulated in both MSC clusters, the mature contractile SMCs, stressed SMCs, contractile SMCs, stressed fibroblasts, fibromyocytes, activated fibroblasts, and intermediate SMCs. *LTBP2* was also downregulated in mature contractile SMCs, contractile SMCs, stressed fibroblasts, fibromyocytes, and activated fibroblasts.

Finally, downstream targets of TGF-β signaling were evaluated (Figure 6D). *COL1A1* and *COL1A2* were upregulated in MFS, mostly in quiescent fibroblasts, fibromyocytes, and stressed fibroblasts. In contrast, *CTGF* expression was generally decreased in SMCs and fibroblasts. However, this difference was not statistically significant. *SERPINE1* was significantly downregulated in fibromyocytes and stressed fibroblasts and upregulated in stressed SMCs.

### 3.10. Other Key Pathways in Non-Immune Cells in MFS

**Figure 7:**
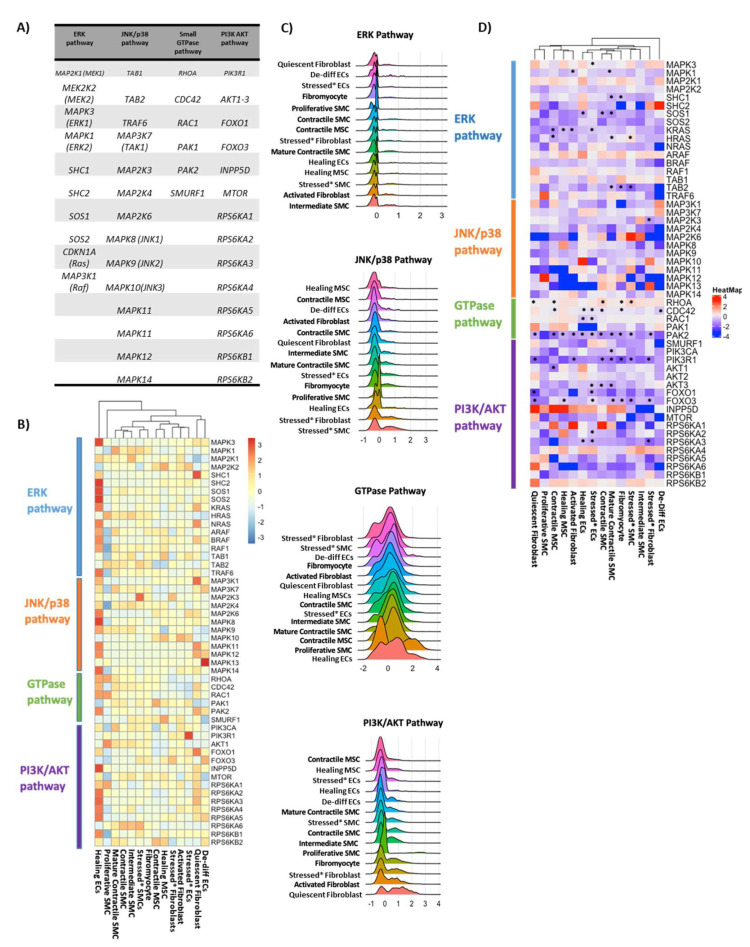
Expression of genes involved in the TGF-β pathway. (**A**) Genes studied in each non-canonical pathway. (**B**) Expression profiles of the different genes involved in non-canonical TGF-β signaling in non-immune cells. (**C**) Module score of genes involved in each non-canonical TGF-β pathway. In all figures, the clusters are ordered based on expression levels with the highest level first. (**D**) Differential expression of genes involved in the non-canonical TGF-β pathway in MFS compared to control tissues. Data are visualized as log2FC in MFS compared to control. * Clusters with significant differential expression (adjusted *p* value < 0.05). ECs, endothelial cells; MSC, mesenchymal stem cells; SMC, smooth muscle cells.

We evaluated the differential expression of genes involved in the non-canonical TGF-β pathways in MFS compared to control tissues to determine whether these pathways are activated by upregulation of TGFB1 in MFS tissues (Figure 7). We have provided a list of genes within each pathway (Figure 7A), their general expression profiles within each pathway (Figure 7B), and the highest expression scores of genes involved in the pathways (Figure 7C). The ERK pathway was most highly expressed in the intermediate SMCs and activated fibroblasts, the JNK pathway in the stressed SMCs and fibroblasts, the GTPase pathway in the healing ECs and de-differentiated proliferative SMCs, and the PIK3/AKT pathway in the fibroblast clusters.

Differential gene expression is shown in Figure 7D. Within the ERK pathway, ERK-1 (*MAPK3*) was downregulated in MFS compared to control samples in stressed ECs. ERK-2 (*MAPK1*) was downregulated in activated fibroblasts and mature contractile SMCs. Several genes upstream of ERK, including *SHC1*, *SOS1*, and *Ras* (*KRAS*), were downregulated in MFS across multiple clusters.

No genes within the JNK/p38 pathway were significantly differentially expressed in MFS compared to controls, except for *MKK3* (*MAP2K3*), which was downregulated in MFS samples in stressed fibroblasts.

Within the small GTPase pathway, *RHOA* was downregulated in quiescent fibroblasts and contractile MSCs and upregulated in contractile SMCs, fibromyocytes, and stressed SMCs. Other genes involved in the GTPase pathway were downregulated in MFS compared to control samples. *CDC42* was downregulated in contractile MSCs, all ECs, contractile SMCs, and fibromyocytes. *PAK2* was downregulated across most clusters.

Finally, we evaluated the PI3K/AKT pathway. Although we previously showed that AKT-2 plays a protective role in the aorta and identified decreased AKT-2 protein levels in sporadic aortic aneurysm tissue [39], in the present study we found no differential expression of *AKT1* or *AKT2* in SMCs or fibroblasts in MFS compared to controls. However, we did observe changes in other genes in the pathway. *AKT3* was downregulated in stressed ECs, contractile SMCs, and mature contractile SMCs. *PIK3CA*, a subunit of PIK3, was downregulated in mature contractile SMCs, and *PIK3R1* was downregulated across most SMC and fibroblast clusters.

## 4. Discussion

In this study, we used the precision of single-cell analysis to compare the transcriptome profile of the aortic wall in patients with MFS and control tissues. We aimed to better understand the factors driving aortic disease in MFS, and we hypothesized that cell-specific changes in cell signaling and development due to FBN1 mutations in MFS may lead to dysfunctional non-immune cells. Here, we highlighted the phenotypic continuum between fibroblasts and SMCs in aortic wall tissue and identified an increased proportion of de-differentiated, proliferative SMCs in MFS tissues, potentially due to a decreased response to TGF-β in MFS compared to control tissues.

Historically, SMCs were believed to undergo a “phenotypic switch” from a contractile phenotype to a synthetic phenotype that produced more ECM proteins. This switch was associated with the advancement of cardiovascular disease. However, more recent studies have supported the idea of a spectrum of SMC phenotypes rather than two distinct groups, and this idea has been expanded with the development of single-cell analysis. Using scRNA sequencing on coronary artery tissue, Wirka et al. identified modulated SMCs (fibromyocytes) that showed a spectrum of ECM production with cells expressing progressively increasing levels of ECM genes as they moved farther from traditional contractile SMCs [22]. Pedroza et al. also identified a spectrum of SMC phenotypes using single-cell analysis of an MFS aortic root SMC line. They found significant upregulation of ECM genes in modulated SMCs in MFS tissue compared to control and an increased proportion of ECM-expressing SMCs in MFS [6]. The same group later showed gradual phenotype change across SMCs and modulated SMCs in the aortic root/ascending aorta in aneurysmal *Fbn1^C1041G/+^* and non-aneurysmal control mice [40].

Our data from single-cell analysis of the aortic wall support an inverse relationship between the expression of contractile and ECM genes that is not binary but that progressively changes within cell clusters not only between SMCs, but even from quiescent fibroblasts to mature contractile SMCs. These findings support the idea of a phenotypic continuum encompassing multiple clusters in addition to the fibromyocytes described by Wirka et al. [22] and align with the scRNA sequencing studies of SMCs in mice by Pedroza et al. [6,40]. Along this continuum between fibroblasts and SMCs, we identified a cluster of de-differentiated, proliferative SMCs that was predominantly composed of cells from MFS tissue and was, in fact, the largest cluster in MFS tissue. Immunofluorescence studies also supported this finding, with increased CCND1^high^ SMCs in aneurysmal ascending aortic tissue in patients with MFS compared to controls. This may represent a cluster of SMCs that has either de-differentiated in response to a stimulus such as mechanical stress or cytokine signaling or did not have the opportunity to differentiate. A modulated SMC cluster unique to MFS has been identified by Pedroza et al. in *Fbn1^C1041G/+^* mice and in a human sample [40], although whether these clusters are similar requires further analysis.

In our evaluation of contractile gene expression, *ACTA2* and *TAGLN* were upregulated in MFS compared to control tissue in many SMC clusters. However, *MYOCD*, which encodes a transcription factor associated with vascular SMC differentiation, was downregulated in mature contractile SMCs and contractile SMCs. Additionally, we showed that *MYH11*, a gene associated with a more mature contractile phenotype, was downregulated in MFS compared to controls in mature contractile SMCs, contractile SMCs, fibromyocytes, and de-differentiated, proliferative SMC clusters. These findings differ from those of Pedroza et al. [6], which showed increased *MYOCD* and *MYH11* in the aortic media in the aortic root of MFS patients by RT-PCR and may reflect SMC-specific differential gene expression within the aortic wall in aneurysmal disease in MFS. The discrepancy may be due to variations in patient data, sample preparation, and gene detection and highlight the complexity of the mechanisms underlying the diverse patterns of alterations of different contractile genes. Further research is needed to fully understand these pathways. In evaluating genes involved in ECM maintenance within SMCs, we noted significant upregulation of *COL1A1/2* and *ELN* in MFS fibromyocytes, which is the cluster of modulated SMCs moving in a fibroblast direction from a traditional contractile phenotype. When combined with our finding of increased de-differentiated, proliferative SMCs in MFS, these results suggest that SMCs are less differentiated toward a mature contractile phenotype in MFS tissues than in control tissues.

Decreased differentiation of vascular SMCs to a contractile phenotype may be associated with aortic disease [41]. Although several pathways may modulate SMC differentiation, the TGF-β signaling pathway is of interest in MFS. Previous studies have shown an increase in contractile genes, including *ACTA2*, *MYH11*, and the transcription factor *MYOCD*, in MFS compared to control tissues in both whole tissue and vascular SMC cultures [5,6]. In addition, the expression of collagen genes I and III was elevated in both whole tissue and vascular SMC culture in tissues from patients with MFS. In one study [5], these increases in collagen and contractile gene expression were mitigated by pharmacological blockade of TGF-β.

Our finding of decreased expression of *MYH11* and *MYOCD* in contractile SMCs and increased expression of *COL1A1/2* and *ELN* in fibroblasts and fibromyocytes in MFS tissue, which suggests decreased SMC differentiation in MFS compared to control tissue, may be due to the downregulation of canonical TGF-β signaling in SMCs in MFS samples. In mature contractile SMCs, the expression of TGF-β receptors TGFBR1, TGFBR2, *BMPR1A*, and *BMPR2* was downregulated in MFS compared to control samples on single-cell analysis. The expression of *TGFBR2* was also downregulated across other SMC clusters in MFS samples, including contractile SMCs, intermediate SMCs, and fibromyocytes. Decreased levels of TGF-β receptor II in SMCs were also supported by immunofluorescence studies. This finding implies a decreased ability of SMCs to respond to TGF-β ligands through the canonical pathway and may indicate an overall decreased stimulus to differentiate towards a mature contractile phenotype. Despite this downregulation of TGF-β receptors, we did identify upregulation of TGFB1, consistent with previous studies. Total plasma TGF-β1 in both mice and humans has been shown to be increased in MFS, and the level of TGF-β has been associated with disease severity [35,42,43,44,45]. Additionally, TGF-β1 was increased in aneurysmal tissue in patients with MFS [45]. In our single-cell data, when immune and non-immune cells were combined to mimic samples for whole tissue RT-PCR or immunoblotting, MFS tissue showed a higher expression of TGFB1 than control tissue, despite downregulation of other factors in the TGF-β pathway. In evaluating the cell-specific differential expression of genes involved in the TGF-β pathway, we found that two particular clusters of fibroblasts mainly drive this finding: activated and quiescent fibroblasts. This indicates that the increase in TGF-β previously described [35,42,43,44,45] is due to an increase in transcription in aortic fibroblasts and not solely overactive signaling secondary to dysfunctional sequestration. In fact, tissue levels of TGF-β1 identified on immunofluorescence were roughly equal in MFS and control samples, indicating a possible disconnect between the increased *TGFB1* RNA and its active ligand form.

Activated fibroblasts have multiple properties that suggest their importance in the aortic wall. Their gene expression patterns indicate involvement in angiogenesis, SMC differentiation, and immune signaling. In addition, activated fibroblasts express the highest level of *NOTCH3*, a gene necessary for early arterial development [19,33]. Later in development, *NOTCH3* appears to be upregulated in response to injury and to continue to modulate SMC phenotype [19,46]. Previous studies have shown that activated fibroblasts may be beneficial in blood vessel healing but are potentially pathologic if overly proliferative [47]. In the vascular wall adventitia, activated fibroblasts contribute to increased release of inflammatory cytokines, resulting in recruitment of monocytes and leading to atherosclerosis and aortic aneurysms [48]. In mice, these fibroblasts are stimulated by angiotensin II [48]. In our study, although these activated fibroblasts were not markedly increased in MFS compared to controls, they appeared to be highly involved in collagen production and vascular healing after injury. Additionally, *TGFB1* expression was most highly correlated with genes associated with angiogenesis, remodeling, and proliferation. Together, these findings indicate that activated fibroblasts play a critical role in responding to aortic stress, suggesting the need for further study in MFS patients.

To better understand the paradoxical upregulation of TGFB1 and downregulation of TGF-β receptors, we evaluated downstream targets of TGF-β signaling. Collagen I and III were upregulated in MFS in quiescent fibroblasts, stressed fibroblasts, and fibromyocytes, suggesting upregulation due to the increase in TGFB1. However, *CTGF* expression was decreased (although non-significantly) in MFS samples in all SMCs and activated fibroblasts, and *SERPINE1* was either downregulated or not differentially expressed. Additionally, TGF-β receptors and downstream SMAD genes were not correspondingly upregulated. This suggests that the upregulation of collagen may be due to activation of alternative pathways. Collagen upregulation in the aortic media may contribute to increased arterial stiffness and progression of aortic aneurysm [45]. Collagen deposition, particularly in the aorta adventitia, may be a response to increased mechanical force (such as hypertension) [49]. Dysfunctional SMC elastic laminae connections caused by the *FBN1* mutation may result in altered mechanostimulation, leading to increased collagen deposition in the aortic wall [50]. Supporting this, we noted the upregulation of *RHOA* in several SMC clusters, which has been shown to be involved in maintaining mechanical homeostasis [51].

One potential explanation for the paradoxical upregulation of *TGFB1* without other markers of increased TFG-β signaling is that *TGFB1* gene expression does not correlate with active TGFβ-1 protein levels. This explanation is supported by our immunofluorescence data showing similar levels of TGFβ-1 in MFS and control tissues. It is possible that the inactive form of the TGFβ-1 protein is produced but cannot be stored in the latent complexes because of the FBN1 mutations and, thus, is not cleaved and activated. In our study, downregulation of both LTBP1 and LTBP2 in SMCs, fibroblasts, and MSCs in MFS supports the decreased formation of latent TGF-β complexes. TGF-β is released in an inactive form and then activated once it is part of the latent TGF-β binding complexes [51]. Antibodies to LTBP1 can inhibit activation of latent TGF-β complexes [52].

We evaluated the non-canonical TGF-β pathway to determine if the upregulated TGFB1 in MFS exerted its effect through alternative methods. TGF-β has been hypothesized to produce different effects when activating the canonical versus the non-canonical TGF-β pathways. Loss of SMAD4, a canonical TGF-β pathway mediator, has been shown to exacerbate the progression of thoracic aortic aneurysm and dissection in mouse MFS models, whereas inhibition of non-canonical TGF-β signaling, mediated through ERK, JNK, and p38, has been shown to protect against progression of thoracic aortic disease [53]. We did not see a significant difference in the expression of most of the genes involved in the non-canonical TGF-β pathways. This finding suggests that in advanced aneurysmal disease in patients with MFS, neither canonical nor non-canonical TGF-β signaling is increased in non-immune cells.

Our study has several limitations. First, the sample size of this pilot study is small. Additional studies with larger sample sizes are needed to confirm our findings. Additionally, most cells in the SMC clusters were from control tissues. This may be due, in part, to decreased SMCs seen in aneurysmal tissues that have become replaced by collagen in advanced disease. We addressed this issue by comparing cluster proportions between MFS and controls and normalizing data to cell count in each cluster. Nevertheless, greater representation of control cells within SMC clusters may have resulted in higher control SMC cluster resolution; thus, control data may have become the “blueprint” for the SMC phenotypic spectrum. Although this may mask smaller differences between SMC clusters in MFS, overall higher cell counts improve our understanding of the phenotypic spectrum throughout the aortic wall. Future samples from MFS patients may greater inform differences in SMC cluster distribution in MFS. Discrepancy between our single-cell and immunofluorescence results showed the increased transcription of TGFB1 without a marked increase in the active ligand. This finding needs further study to identify the factors involved in this discrepancy. We acknowledge that the control tissue has a higher expression of the early response genes shown [24] to be most affected by temperature and processing time. This is thought to be due to differences in how the samples are obtained and processed rather than inherent differences in the tissue of MFS patients and controls. For this reason, we have avoided drawing conclusions based on differential expression of these early response genes. Additionally, differences between control tissue and MFS patient tissue may not account for sex-based differences as our patient cohort was not sex matched. Finally, this study was conducted in patients with advanced aneurysmal disease, and these findings may not be representative of the expression level of TGF-β pathway genes throughout aneurysm development.

In conclusion, in a single-cell transcriptome analysis on human ascending aortic aneurysm tissue in patients with MFS and on tissue from controls, we identified an extensive phenotypic continuum of fibroblasts and SMCs. One cluster, the de-differentiated proliferative SMCs, was markedly increased in MFS compared to control tissue, which may indicate changes in signaling pathways driving SMC differentiation in MFS, such as the TGF-β pathway. Despite an upregulation of TGFB1 in fibroblast clusters in MFS, our data support an overall decrease in canonical and non-canonical TGF-β signaling in advanced aneurysmal disease in MFS and suggest this decrease, particularly in SMCs, may be responsible for an overall less-differentiated contractile SMC phenotype. In future studies, we plan to identify cell populations and differential gene expression within immune cells in MFS and to continue the enrollment of tissue samples to study sex-based differences in aortic aneurysm development in MFS patients.

## Data Availability

The data underlying this article will be made available in the GEO (Gene Expression Omnibus) database.

## Supplementary Materials

The following are available online at https://www.mdpi.com/article/10.3390/genes13010095/s1: Figure S1: Individual sample data; Figure S2: Overall gene expression levels in non-immune cells; Figure S3, General cluster identification in immune and non-immune cells in combined samples; Figure S4, Identification of SMC phenotype; Figure S5, Identification of fibroblast phenotype; Figure S6, Identification of endothelial cell and mesenchymal stem cell phenotype; Figure S7, Identification of immune-like non-immune cells; Figure S8, Cell-cell signaling within the TFG-β pathway; Figure S9, Differential expression of genes involved in the control of TFG-β signaling; Table S1, Patient sample information; Table S2, Antibodies used in immunofluorescence; Table S3, Genes associated with *TGFB1* in “activated fibroblasts”; Table S4, Genes associated with *TGFB1* in “quiescent fibroblasts”.
