# Supplementary material for: Single-Cell Analysis of Aneurysmal Aortic Tissue in Patients with Marfan Syndrome Reveals Dysfunctional TGF-β Signaling
Source: Genes (Basel). 2021 Dec 30;13(1):95. doi: 10.3390/genes13010095 (PMC8774900; doi:10.3390/genes13010095)
Supplement: Supplementary file 1 [file genes-13-00095-s001.zip › genes-1458406-supplementary.pdf]

SUPPLEMENTAL MATERIAL

Single-cell Analysis of Aneurysmal Aortic Tissue in Patients With Marfan Syndrome Reveals Altered Smooth Muscle Cell Differentiation and Dysfunctional TGF-β Signaling

Ashley Dawson, MD, Yanming Li, PhD, Pingping Ren, MD, PhD, Hernan G. Vasquez, PhD, Chen Zhang, MD, Waleed Ageedi, MD, Alon R. Azares, MS, Aladdein Mattar, MD, Mary Burchett Sheppard, MD, Hong S. Lu, MD, PhD, Joseph S. Coselli, MD, Lisa A. Cassis, PhD, Alan Daugherty, PhD, Ying H. Shen, MD, PhD, Scott A. LeMaire, MD

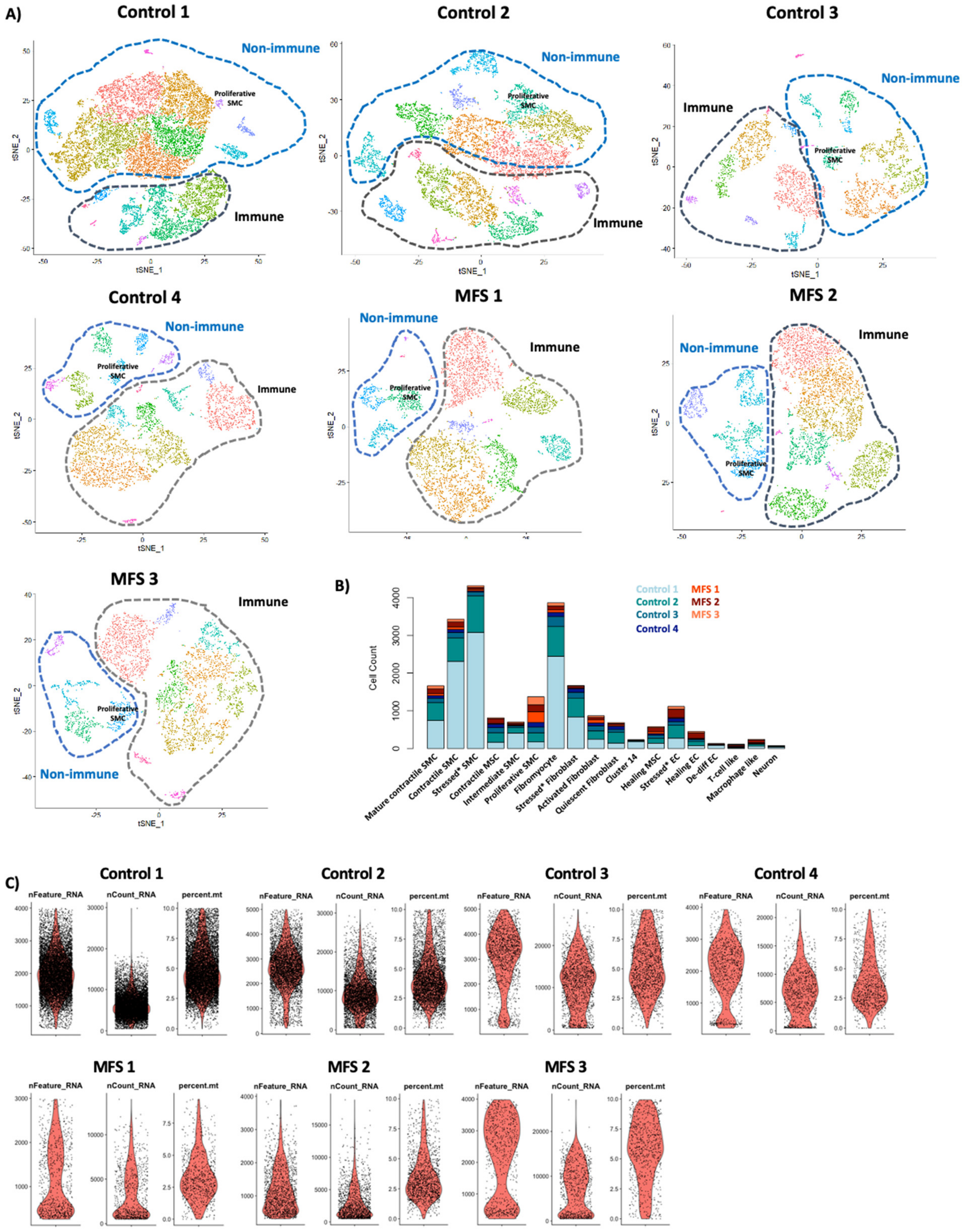

Figure S1. Individual sample data. A, Individual tSNE plots for each sample. The non-immune cells were identified and extracted. The de-differentiated, proliferative SMCs

are indicated in each sample. **B,** Cell count in each cluster from each sample. Control tissues had increased numbers of SMCs compared to MFS tissues**. c,** Quality control measures including the distribution of features (genes), RNA counts, and percent mitochondria genes (percent.mt) for the non-immune cells of each sample.

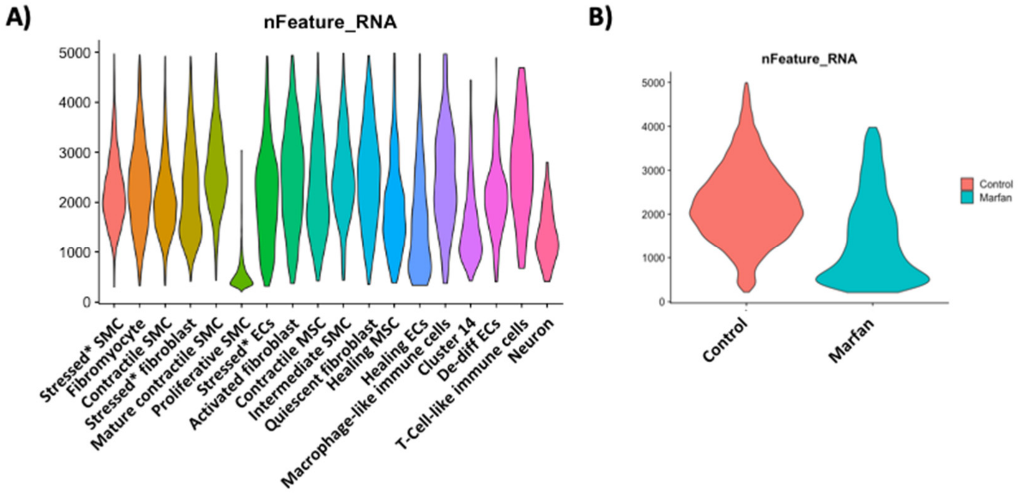

Figure S2. Overall gene expression levels in non-immune cells. A, De-differentiated, proliferative SMCs have a relatively low level of gene expression compared to other clusters. **B**, Gene expression levels are lower in Marfan samples in general.

A)

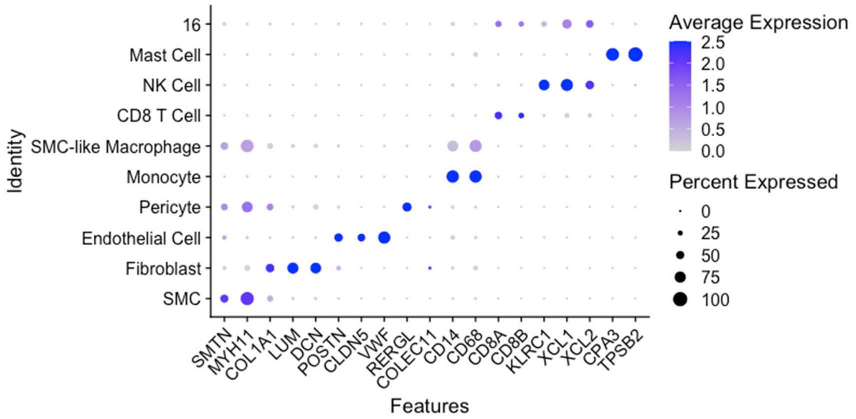

B)

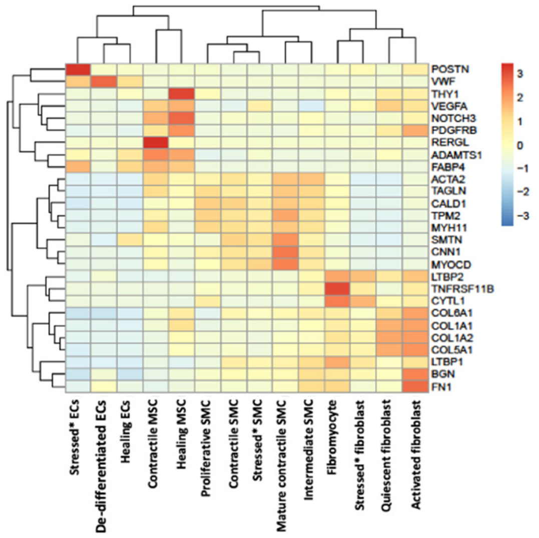

Figure S3: General cluster identification in immune and non-immune cells in combined samples. A, Common marker genes were used to identify clusters. **B,** Correlation analysis showing similar types of non-immune cells in combined MFS and control tissues based on gene expression patterns.

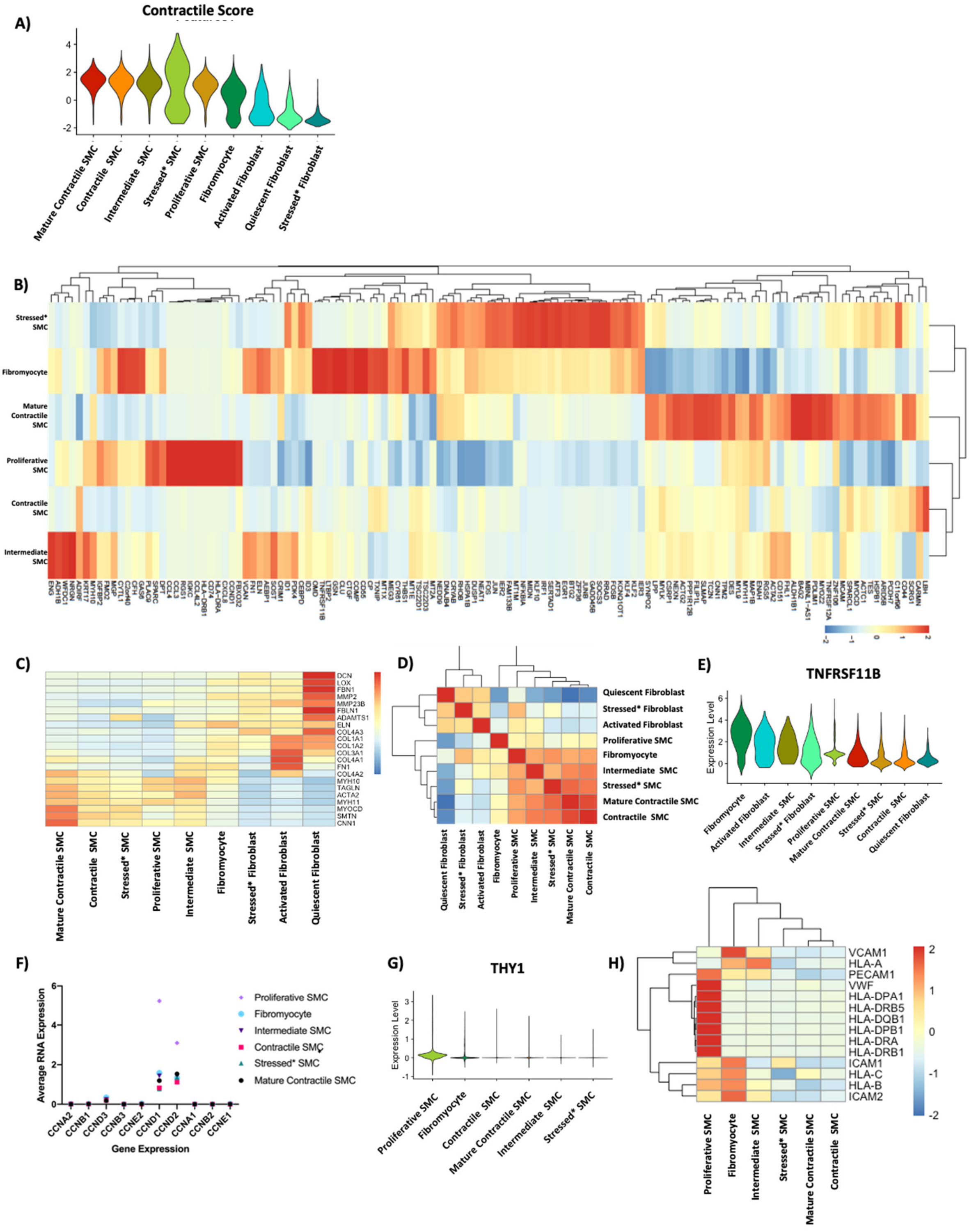

Figure S4. Identification of SMC phenotype. Heatmaps are scaled by row. **A**, Contractile composite scores of RNA expression of *SMTN*, *MHY11*, and *CNN1* used to identify the mature contractile SMCs. Clusters are ordered from highest to lowest

expression from left to right. **B**, Top 20 upregulated and downregulated genes within each SMC-like cluster compared to mature contractile SMCs. **C**, Expression of functional genes involved in contraction and ECM production in SMCs and fibroblasts. **D**, Correlation analysis between fibroblasts and SMCs. **E**, Expression of *TNFRSF11B*, a marker for fibromyocytes, in fibroblasts and SMCs. **F**, Expression of cyclin genes. **G**, Expression of THY-1 (CD-90), a marker of stem cell-like cells. **H**, Expression of inflammatory genes including those for HLA-I and HLA-II and for interleukins. *Stressed cells likely represent technical artifact.

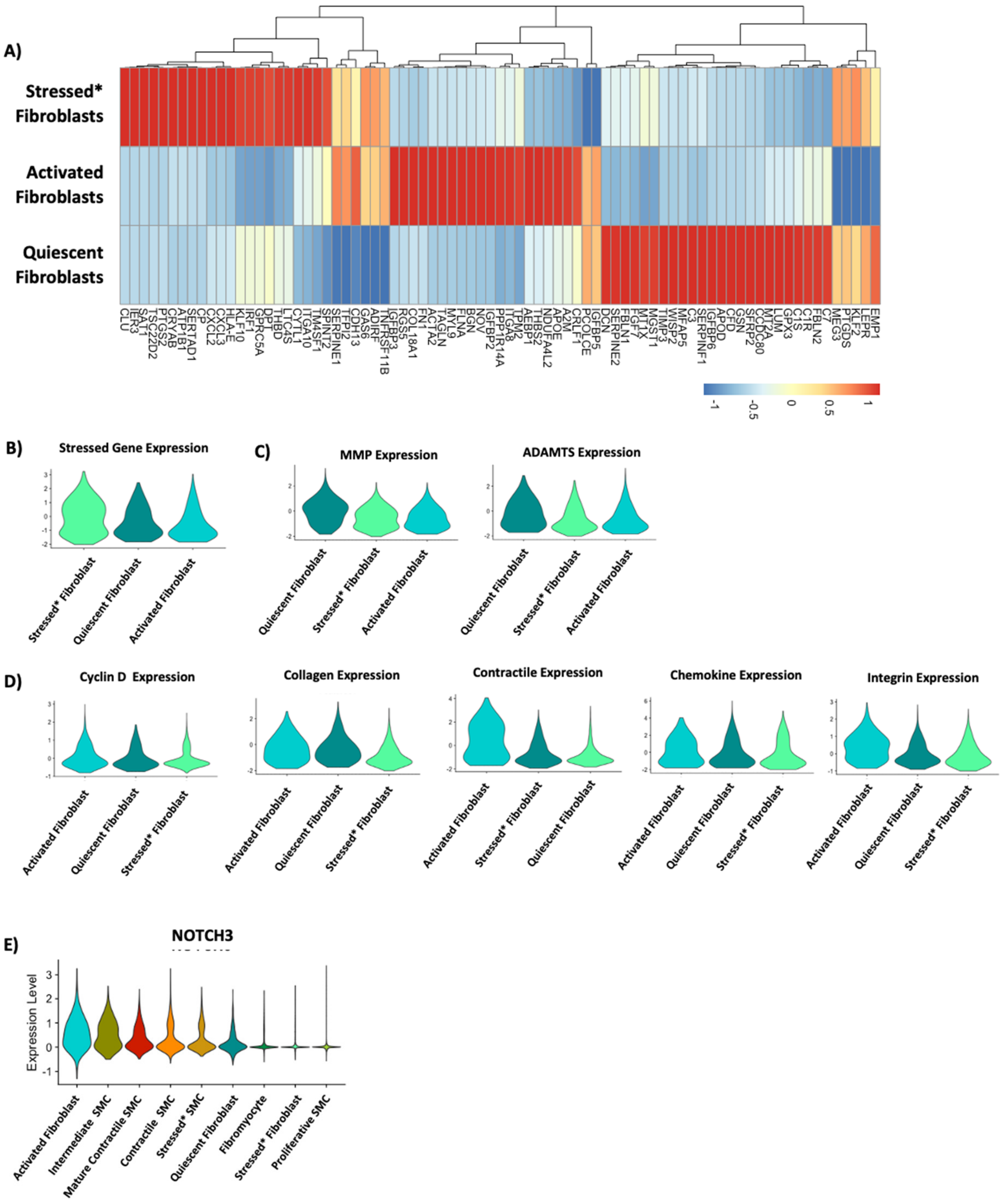

Figure S5. Identification of fibroblast phenotype. **A**, Top 20 upregulated and downregulated genes between fibroblast clusters. Heatmap is scaled by row. **B**, Stress response genes area highest in the stressed fibroblasts (*consistent with technical artifact secondary to tissue processing). **C**, Matrix metalloproteinase (MMP) and ADAMTS gene expression is highest in quiescent fibroblasts. **D**, Expression of cyclin D, collagen, contractile, chemokine, and integrin genes are highest in activated fibroblasts.

E, Expression of NOTCH3, a gene involved in modulation of SMC phenotype and angiogenesis and cell differentiation in the injury response, is highest in activated fibroblasts.

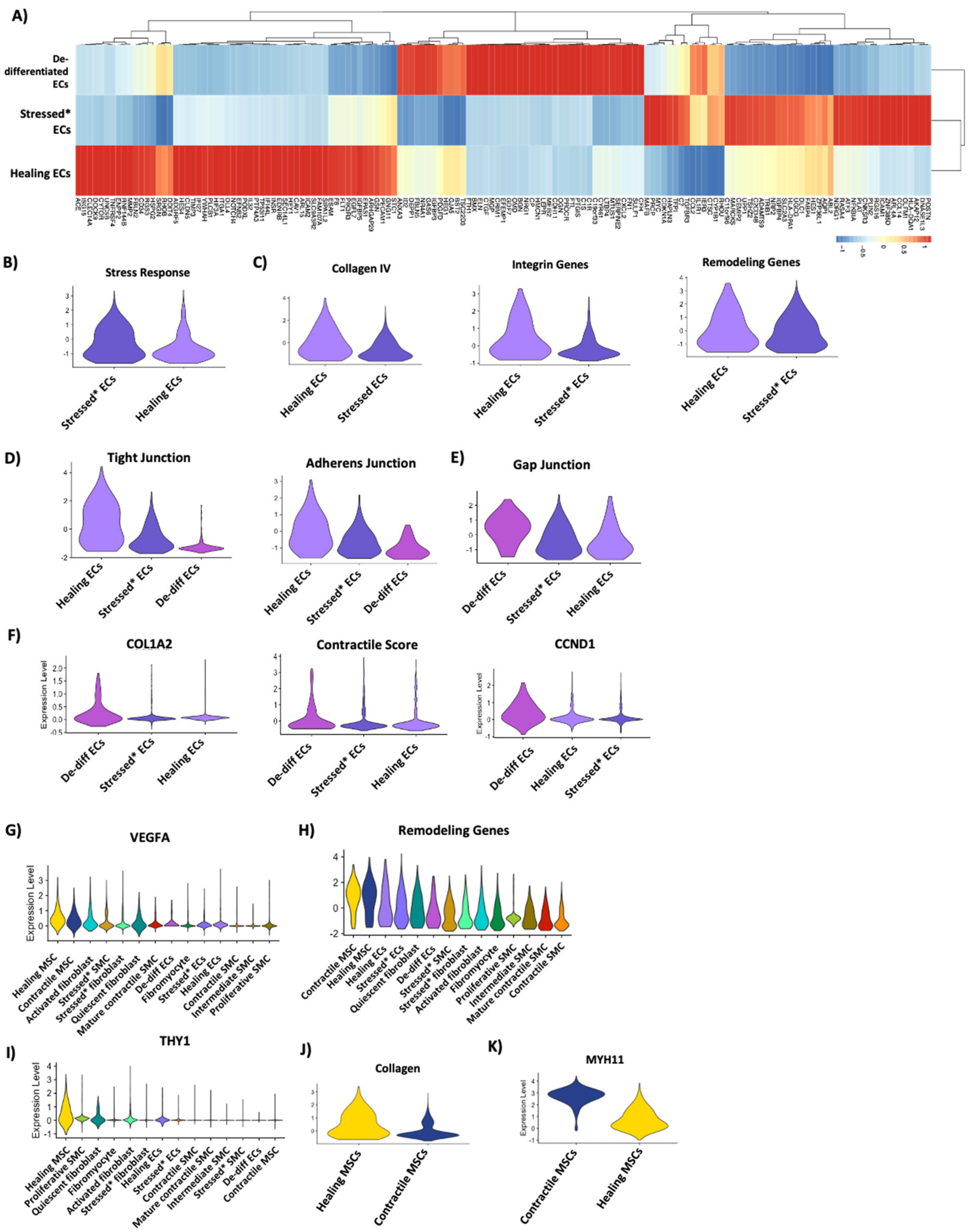

Figure S6. Identification of endothelial cell and mesenchymal stem cell phenotype. **A,** Top 20 upregulated and downregulated genes between each endothelial cell (EC) cluster. Heatmap is scaled by row. **B,** Higher composite score of stress response genes

ATF3, JUN, JUNB, FOS, and FOSB in the stressed ECs, (*consistent with technical artifact secondary to tissue processing). **C,** Higher expression of collagen, integrin, and remodeling genes in healing ECs. **D,** Healing ECs most involved in tight and adherens junctions. **E,** De-differentiated ECs with higher gap junction gene expression. **F,** De-differentiated ECs with higher collagen I (COL1A2), contractile module score, and cyclin D1 (CCND1) expression. **G,** Overall highest composite score of vascular endothelial growth factor-A (VEGFA) in the mesenchymal stem cell (MSC) clusters. **H,** Overall highest expression of remodeling genes in the MSC clusters. **I,** Overall highest expression of THY1 in healing MSCs. **J,** Higher composite score of collagen genes in healing MSCs. **K,** Higher composite score of the contractile gene MYH11 in contractile MSCs.

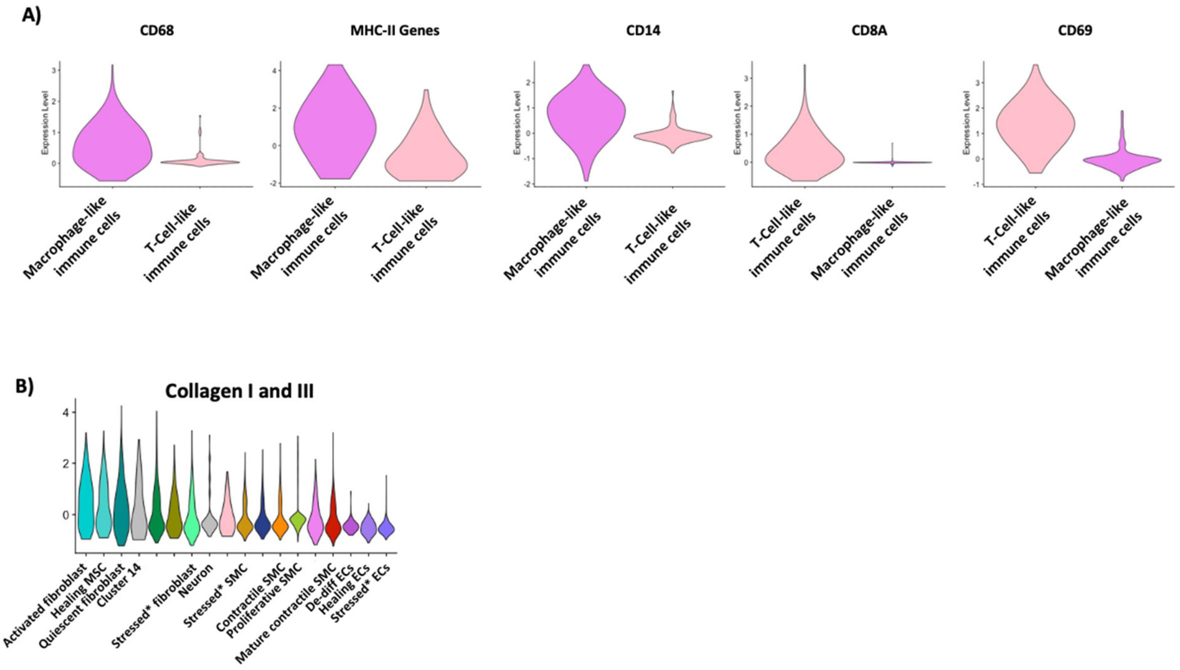

Figure S7. Identification of immune-like non-immune cells. A, Higher expression levels of macrophage markers in the macrophage-like non-immune cells showing CD68, antigen-presenting MHC-II genes, and CD14. Higher expression of T-cell genes in the T-cell-like non-immune cells showing CD8A and CD69. **B,** Collagen I and III expression in immune-like non-immune cells is comparable to smooth muscle cells (SMCs) and higher than that in endothelial cells (ECs).

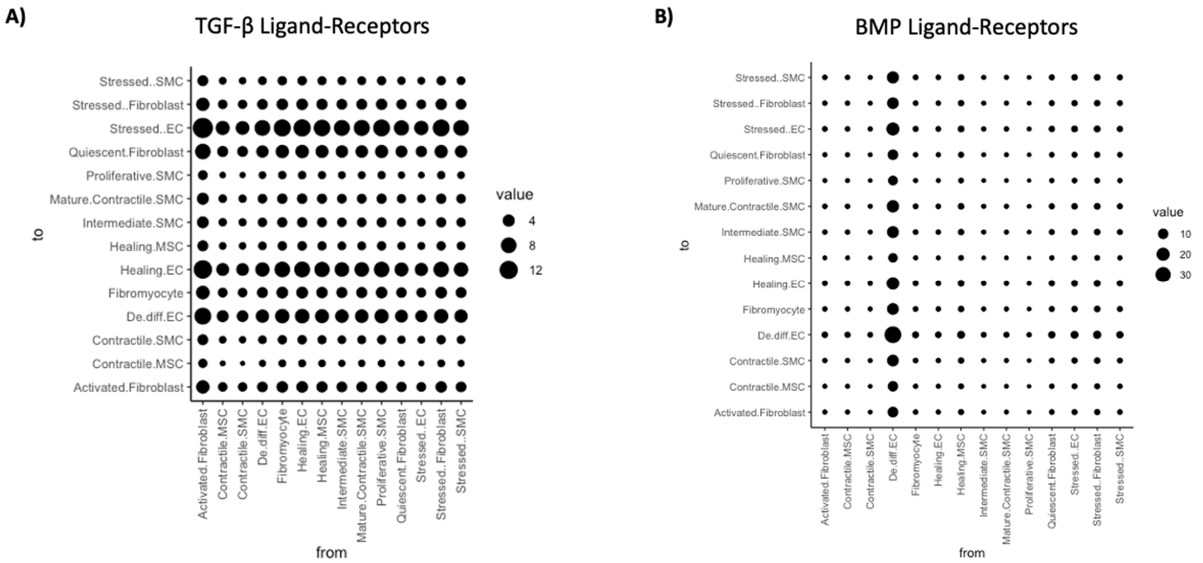

Figure S8. Cell-cell signaling within the TGF-β pathway. A, Junctional analysis of TGF-β ligands and receptors. Ligands include *TGFB1*, *TGFB2*, *TGFB3*. Receptors include *TGFBR1*, *TGFBR2*, *TGFBR3*, *ACVRL1*. **B,** Junctional analysis of BMP ligands and receptors. BMP ligands include BMP 2-7 and receptors include *BMPR1A*, *BMPR1B*, *BMPR2*, *ACVR1B*, *ACVRL1*, *ACVR2A*, *ACVR2B.*

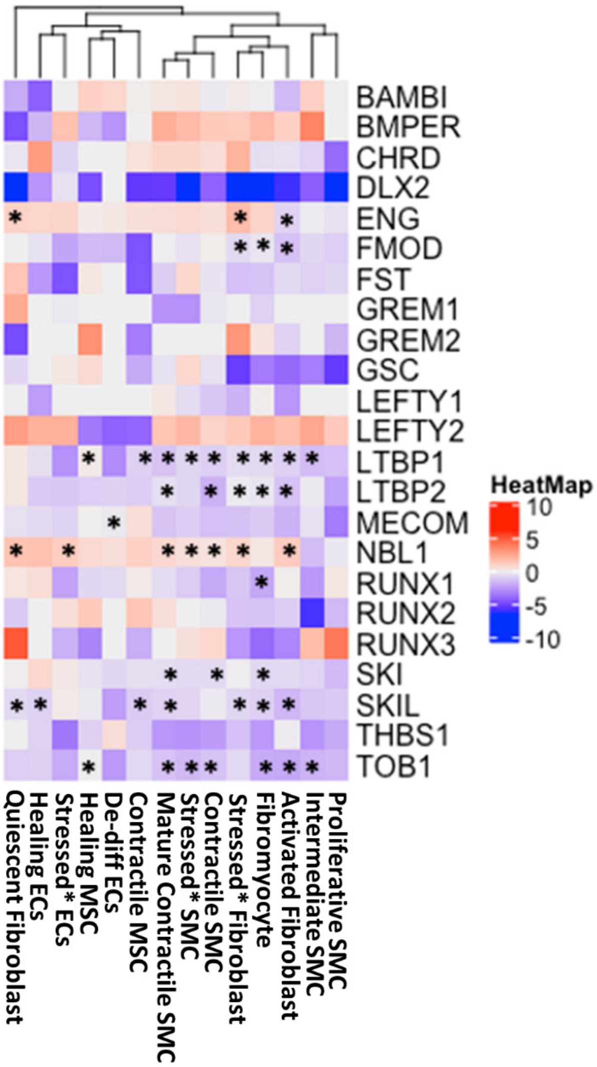

Figure S9. Differential expression of genes involved in the control of TGF-β signaling. Data are visualized as log2FC in MFS compared to control tissues. *Clusters with significant differential expression (adjusted p value <0.05). EC, endothelial cells; MSC, mesenchymal stem cells; SMC, smooth muscle cells.

Table S1. Patient sample information

Sample	Age	Sex	Race	Ethnicity	Aortic Root Diameter (cm)	Asc Aortic Diameter (cm)	Smoking History	Medical History	Genetic History
Control 1 (Recipient)	47	M	Unknown	Unknown	N/A	N/A	Unknown	NICM, pHTN, DM, HTN, ESRD	N/A
Control 2 (Donor)	44	M	Unknown	Unknown	N/A	N/A	Past	DM	N/A
Control 3 (Donor)	38	M	White	Non-Hispanic	N/A	N/A	Yes, 20 pack years	None	N/A
Control 4 (Donor)	23	M	White	Non-Hispanic	N/A	N/A	Never	None	N/A
MFS 1	40	M	White	Non-Hispanic	4.6, previously repaired	5.3	Past	CHF	Diagnosed with MFS at 19 years old at OSH, kyphosis, scoliosis. FHx: brother and father with MFS
MFS 2	23	F	White	Non-Hispanic	4.2	3	Past	HTN, CHF	Diagnosed with MFS at 5 years old at OSH, pectus excavatum. FHx: mother, cousin with MFS
MFS 3	38	F	White	Hispanic/Latino	6.1	3.6	Never	HLD, HTN, CHF	FHx: At least two brothers with MFS, one brother with pathologic variant on *FBN1*

Ascending (AS), congestive heart failure (CHF), diabetes mellitus (DM), end-stage renal disease (ESRD), family history (FHx,), hyperlipidemia (HLD), hypertension (HTN), Marfan syndrome (MFS), nonischemic cardiomyopathy (NICM), outside hospital (OSH), pulmonary hypertension (pHTN).

Table S2. Antibodies used in immunofluorescence

Antibody	Company	Catalog Number
TGFB1	Abcam	ab92486
TGF-beta receptor II	Cell Signaling	79424S
Cyclin D1	Cell Signaling	2978S
Anti-TAGLN/Transgelin	Abcam	ab10135

Table S3. Genes associated with *TGFB1* in "activated fibroblasts"

Gene	Correlation	Function
TGFB1	1	
MMP11	0.272426	Remodeling
PTP4A3	0.246508	Proliferation
PLXDC1	0.24615	Angiogenesis
SPARC	0.242422	Extracellular matrix structure
BRK1	0.222364	Endocytosis trafficking
RGS5	0.218113	Hypoxia inducible, endothelial apoptosis
DERL3	0.216915	Degradation of misfolded proteins
TGFBI	0.214094	Proliferation
EGFL7	0.212958	Proliferation, vasculogenesis
CPE	0.211162	Hormone/signaling secretion

Table S4. Genes associated with *TGFB1* in "quiescent fibroblasts"

Gene	Correlation	Function
TGFB1	1	
ZCRB1	0.187697	RNA splicing
TMX1	0.186799	ER protein folding, redox reactions
GSDMD	0.185679	Pyroptosis
WDR54	0.18448	Regulation of ERK signaling
TGFBI	0.182359	Proliferation
TAGLN2	0.180823	Smooth muscle contraction
OAZ2	0.179703	Induces inhibitory SMAD6
EIF3L	0.177385	Protein synthesis
HEYL	0.176706	Transcription factor
RPL13A	0.176562	Ribosomal protein

Endoplasmic reticulum (ER).
